# Development and Validation of the Test of Orthorexia Nervosa (TON-17)

**DOI:** 10.3390/jcm10081637

**Published:** 2021-04-12

**Authors:** Aleksandra M. Rogowska, Aleksandra Kwaśnicka, Dominika Ochnik

**Affiliations:** 1 Institute of Psychology, Faculty of Social Sciences, University of Opole, 45-052 Opole, Poland; aleksandra.kwasnicka@uni.opole.pl; 2 Faculty of Medicine, University of Technology, 40-555 Katowice, Poland; dominika.ochnik@wst.pl

**Keywords:** addictive behavior, disordered eating, obsessive-compulsive disorder, orthorexia nervosa, self-report questionnaire, validation study

## Abstract

This study aims to develop and validate a new self-report questionnaire to measure orthorexia nervosa (ON). Based on a current review of the scientific literature and interviews with people at risk of orthorexia, 40 items were selected to test orthorexia nervosa (TON-40). A total sample of 767 individuals (*M* = 26.49, *SD* = 9.66, 56.98% women) participated in the study. The exploratory factor analysis (EFA), confirmatory factor analysis (CFA), and composite construct analysis (CCA) were performed to find an appropriate model of sufficient reliability and validity and stable construction. Convergent validation was performed regarding the correlation of the TON-17 with another measure of ON (ORTO-15), eating disorders (the EAT-26 and DEAS), healthy behavior (the HBI), quality of life (the Brief WHOQOL), physical health (the GRSH), anxiety (the GAD-7), depression (the PHQ-9), and obsessive-compulsive disorder (the OCI-R). Gender, Body Mass Index (BMI), and the medical reasons for a restrictive diet were also examined. As a result of the structural analyses, the number of items was reduced from 40 to 17. The best fit indices of the TON-17 were found for the hierarchical bi-factor model, with three lower-order factors (Control of food quality, Fixation of health and healthy diet, and Disorder symptoms) and one general higher-order factor (Orthorexia). According to the 95th percentile method of estimation, the prevalence of ON was 5.5% for the TON-17 total score. The TON-17 scale and subscales showed good psychometric properties, stability, reliability, and construct validity. The TON-17 indicated a positive relationship with the ORTO-15, EAT-26, DEAS, HBI, OCI-R, GAD-7, and PHQ-9. TON-17 can be considered as a useful tool for assessing the risk of ON.

## 1. Introduction

### 1.1. Characteristics of People with Orthorexia

Orthorexia nervosa (ON) is defined as “a pathological obsession, fixation or preoccupation with healthy food” [1] (p. 1). Although many researchers agree with the above definition, particular approaches to ON vary, highlighting different diagnosis criteria [2], such as weight loss [3], phobic avoidances [4], and the exclusion of food allergies or medical conditions that require restrictive diets [5,6]. Differences in the conceptualization of ON are reflected in the measurement methods, as has been shown in a recent review [1]. So far, ON has not been recognized as a separate disorder in either the Diagnostic and Statistical Manual of Mental Disorders (DSM-5) or the International Statistical Classification of Diseases and Related Health Problems (ICD-10). In particular, no consensus has been found, regarding whether ON should be classified as a single syndrome of an eating disorder or a variance of other syndromes, such as anorexia nervosa (AN), avoidant/restrictive food intake disorder (ARFID), obsessive-compulsive disorder (OCD), obsessive-compulsive personality disorder (OCPD), somatic symptom disorder, illness anxiety disorder, or psychotic spectrum disorders [7,8,9,10,11].

Orthorexia nervosa (ON) may be understood as a trait characterizing a restrictive and avoidant eating behavior and a tendency to pathological obsession and preoccupation with healthy, strictly organic, and biologically pure foods. Moroze et al. [3] described people with ON as those who avoid foods containing genetically modified ingredients, fat, sugar, salt, preservatives, or food including artificial substances, such as pesticides and herbicides. Orthorexic tendencies are associated with special eating behavior features, such as dieting frequency or vegetarian and vegan diets [12]. Research has indicated that dieting is associated with greater ON [13,14]. In particular, vegans and vegetarians are more likely to develop a pathological preoccupation with healthy eating [15,16]. Individuals with ON demonstrate a fixation on healthy food (e.g., derived from organic agriculture) and often introduce rituals to control their daily routine. For example, they can maintain a long time between meals in order to combine certain foods regarding self-imposed rules [5,10]. Individuals with ON are also concerned about the manner and materials used in the preparation of the food. The process of detailed planning, shopping, and preparation of food requires most of their day-time. This may result in the sense of guilt, feelings of nervousness, unhappiness, frustration, or other negative emotions, if some healthy food rules are not fulfilled [17]. Due to numerous dietary restrictions and a lack of trust in food preparation by someone else, individuals with ON usually refuse to eat away from home and avoid social gatherings, leading to social isolation.

It should be noted that early studies on orthorexia were conducted using the Bratman Orthorexia Test (BOT) and ORTO-15. However, these two tools have been widely criticized for their lack of validity and poor reliability, and therefore the results of these studies may be biased or incorrect [14]. Depending on the measurement tool and country of origin of respondents, the prevalence of ON ranged from 1% to 89%, with higher rates (of 35–89%) among artists, healthcare professionals, nutrition students, dietitians, athletes, or Ashtanga yoga practitioners, as suggested review studies [6,18]. However, Dunn et al. [19] have suggested that most research is overestimated, as the assessment tools do not differentiate people with ON from those with healthy eating habits. Varga et al. [6] showed in the review study that the average prevalence rate for ON was 6.9% for the general population. Some recent studies, conducted in various countries, indicate prevalence rate 21% or less using cut-off score for ORTO-15 ≤ 35 [20,21,22], and prevalence between 2.3% and 7.8%, using cut-off score for Düseldorf Orthorexia Scale (DOS) ≥ 30 [23,24,25,26,27].

In the Polish population, ON risk prevalence ranged from 27% to 69% when the ORTO-15 test [28] was applied. Łucka et al. [29] recognized ON risk in 27% of school-age youth (including 65% of undergraduates). Plichta and Jeżewska-Zychowicz [30] found ON risk in 28.7% of college students (32.9% among health-related majors, 23.9% in students of other majors). Stochel et al. [31] showed ON risk in 47% of high-school students, whereas Brytek-Matera et al. [32] demonstrated prevalence rates of 69% in females and 43.2% in male university students. Using the Bratman Orthorexia Test (BOT) [33], Gubiec et al. [34] showed that 33% of nutrition students have ON, 39% were at a high risk of ON, and only 28% did not report ON symptoms. Dittfeld et al. [35] showed that 26% of healthy food fanatics in a sample of non-vegetarians and 30% in vegetarians were at risk of ON, when BOT was used for the ON measurement. In comparison, ON was recognized among less than 1% of participants in the total sample.

Varga et al. [6] demonstrated in their review that some research indicated that among people with ON, men prevailed over women while, in the other studies, the relationship was reversed or statistically insignificant. A systematic review and meta-analytic integration [36] showed that orthorexic tendencies were similar in both genders, but the results varied depending on the instrument in use. ON seems slightly more pronounced in women than in men, but with a small effect size. A recent review [13] has shown that gender is unrelated to ON. Furthermore, ambiguous relationships between ON and Body Mass Index (BMI) have been found in previous research [6,13].

### 1.2. Association of Orthorexia with Health

The persistent disturbance of eating-related behavior may lead to impaired health and psychosocial functioning, including such medical consequences of selective eating as osteopenia, anemia, pancytopenia, hyponatremia, metabolic acidosis, bradycardia, gastrointestinal problems, stomach inflammation, and other diet-induced ailments [6,9]. Brytek-Matera et al. [9] suggested that some clinical features of people with eating disorders (EDs) are shared by people with ON, including pleasure about food and eating, perfectionism, anxiety, and the displacement onto the food of the sense of control one is not able to achieve in their own life. However, individuals with ON focus more on food quality and purity, while people with ED are concerned about the amount and types of food they eat (e.g., sugar or fat is usually excluded from their diet) [37]. Nevertheless, ON seems to represent a phenomenological subtype of restrictive ED [13,38,39,40,41,42].

Strahler [43] showed that ON is positively related to stress, anxiety, and depressive symptoms and negatively related to psychological wellbeing and life satisfaction. A current review [13] has tested the relationship between ON and various demographic variables, psychological traits, and mental health indices. It was confirmed that higher ON is positively associated with perfectionism, obsessive-compulsive traits, psychopathology, poor body image, and body mass controlling for thinness. An inconclusive result was drawn concerning body dissatisfaction and substance use, such as alcohol, tobacco, and drugs [13].

### 1.3. The Current Study

Although several assessment tools have been developed to date, criticism regarding the reliability and validity of existing questionnaires has led to the conclusion that there is a continuous need to create new tools to investigate ON [1,12,13,18,41,44]. A recent systematic review and reliability generalization [1] identified ten scales measuring ON: Body Image Screening Questionnaire (BISQ), Burda Orthorexia Risk Assessment (B-ORA), Bratman Orthorexia Test (BOT), Düsseldorf Orthorexia Scale (DOS), Eating Habits Questionnaire (EHQ), Eating Habits Questionnaire-Revised (EHQ-R), Orthorexia Nervosa Scale (ONS), ORTO-15, Scale to Measure Orthorexia in Puerto Rican Men and Women, and Teruel Orthorexia Scale (TOS). These Orthorexia Nervosa scales’ psychometric properties indicated that the main issues are related to dimensionality and conceptualization [1,2,41]. For example, unstable factor structure and/or item-allocation in particular factors were shown for ORTO-15, EHQ, and DOS [1]. As suggested Valente et al. [41], the validations of BISQ, B-ORA, BOS, and the Puerto-Rican ON scale, were fragmented and often based on specific populations (e.g., university students). Such tools as BISQ, ORTO-15, BOT, and ONS showed weak internal consistency (assessed by the Cronbach’s alpha reliability coefficient). Most questionnaires (i.e., BOSQ, B-ORA, BOT, EHQ, ONS, and the Puerto-Rican ON), did not calculate test–re–test reliability measures [1]. Moreover, the questionnaires differ depending on the concept of ON and different criteria of ON [2,41]. Opitz et al. [1] have suggested focusing on establishing a consensus regarding ON’s conceptualization to determine a measure with robust psychometric properties. Starting from the methodological weaknesses identified by the recent review, Valente et al. [41] have recommended developing a modern re-conceptualization of ON, comprehensive of end-user perspectives; adopting qualitative data collection techniques to gain insights into how to diagnose ON; and actively involving diverse stakeholders for constructing a new tool.

Unless the DSM and ICD manuals establish a clear definition and diagnostic criteria, any orthorexia approach can be equally important to understand the disorder’s nature. At present, there is no consensus whether ON is a type of ED, AN, ARFID, OCD, OCPD, somatic, anxiety, or psychotic spectrum disorder [7,8,9,10,11]. Different approaches to ON’s measurement and structure, and more research of each assessment’s construct validity, can be useful in solving this problem. The replicability and repeatability of some factors can answer questions about the essence and general structure of ON. Therefore, only future research will decide which of the ON scales is more accurate than others. 

This study aims to analyze the psychometric properties and validate a new measure of ON, namely the Test of Orthorexia Nervosa (TON). TON was developed in accordance with Valente et al.’s [41] postulates. It is essential to find a reliable and accurate screening tool that can be considered helpful for the assessment of orthorexia according to the re-conceptualized ON criteria. In particular, the development of orthorexia from healthy to pathological nutrition has never been considered in ON’s existing questionnaires. It should be noted that consistent with Bratman’s current theory, ON has two stages [45]. In the first stage, people decide to eat a healthy diet. Interest in healthy eating does not always become pathological. However, further progress occurs when an individual adopts non-standard nutritional ideas that seem irrational, unscientific, or strange. In the second stage, intensification of the pursuit of ON’s interest is presented in obsessive thinking, compulsive behavior, self-punishment, and escalation of limitations. 

The present study examines the reliability, and also structural, and construct validity of the TON. Construct validity tests the hypothesis about the positive relationship of a new tool with the other measures of ON, eating disorders, disordered eating attitude, vegetarian and pure diets, various dimensions of healthy behavior (including dietary pattern), and three indices of mental health, including obsessive-compulsive disorder, generalized anxiety disorder, and depression. It is also assumed that the TON scores are negatively related to quality-of-life domains and physical health. The ambiguous relationship of ON with gender and BMI is also tested, in this study, using the TON.

## 2. Materials and Methods

### 2.1. Participants

Initially, 786 people approached to participate in the study. Sixteen respondents refused to participate in the research, and 770 people agreed to complete surveys, including 341 individuals using the online form and 429 using the paper-and-pencil version. We excluded three surveys from further analysis, as the missing data exceeded 5% of responses (two in the online form and one in paper-and-pencil). The total number of valid surveys for analysis was 767 (339 in the online version and 428 in paper form). Missing data have been replaced with mean values.

The total sample consisted of 767 people with a mean age of 26 years (*M* = 26.49, *SD* = 9.66), including 437 women (56.98%). All participants were Caucasian Polish citizens living in various regions of the country. We did not control the online group with regards to employment status or studying. The paper-and-pencil sample consisted of soldiers from one unit (*n* = 28, 6.54%), healthcare professionals from one hospital (*n* = 42, 9.81%, including 21 physicians and 21 nurses), and students from three universities (*n* = 358, 73.84%). The study majors were as follows: Psychology (*n* = 133, 31.08%), Cosmetology (*n* = 103, 24.07%), Physical Education (*n* = 92, 21.50%), and Tourism and Recreation (*n* = 30, 7.00%). Table 1 demonstrates the study sample’s demographic characteristics, such as age, height, weight, BMI, gender, place of residence, current diet, the reason for following the diet, and food-related diseases that require a special diet.

### 2.2. Measures

#### 2.2.1. Test of Orthorexia Nervosa (TON)

The Test of Orthorexia Nervosa (TON) was developed in the spring of 2018 as part of a social research seminar as a course for a group of 12 third-year psychology students at the Institute of Psychology at the University of Opole. In the first step, students reviewed quantitative and qualitative studies to seek all ON information, including diagnostic criteria, description of main traits and demographic characteristics of people with ON, and the relationships of ON with the other psychological traits and mental health disorders. Based on this review of the scientific literature on the phenomenon of orthorexia and previous tools to measure ON (e.g., ORTO-15 and BOT), the students prepared several dozen questions in four groups (three students in each sample).

The questions were used to prepare a structured interview (see Supplementary Table S1). Each of the four groups then interviewed a friend who was overly interested in a healthy diet (e.g., often talked about healthy and unhealthy eating, pointed out that certain products must not be eaten, was knowledgeable about organic food, knew quality certificates). The results of the interviews were presented and discussed at the seminar. After a demonstration of the interview results, all 12 students with the lecturer (AMR) participated in preparing the final questions for the TON. The target was to develop a new questionnaire to measure ON, based upon self-experience with qualitative research and a review of previous studies, including Bratman’s [33,45] conceptualizations of ON and current diagnostic criteria [2,3,6,10,18]. A pool of 80 questions describing thoughts and behaviors related to orthorexia was created using the brainstorming technique.

The items reflected attitude towards healthy eating: food restrictions, fear of eating something prepared by others, reluctance to buy or eat food containing preservatives or coloring agents, preoccupation with food ingredients, and experiencing a decline in health or relationships due to being so focused on eating only healthy food. Part of the statements expressed potential behaviors, and other parts represented emotional states and beliefs. Then some questions were democratically eliminated by a majority of the votes. As a result of eliminating repeated items or those weakly related to the essence of orthorexia, in the next stage, 40 items were selected and the content has been improved for clarity and simplicity. The final version of the questionnaire comprised 40 questions (see the TON-40 in the Supplementary Materials). Each item was rated on a five-point Likert-like scale, indicating the degree of compliance with the sentence (1 = Strongly disagree, 2 = Disagree, 3 = Undecided, 4 = Agree, 5 = Strongly agree). The scores derived from all 40 items are summarized, with higher scores indicating a stronger tendency towards orthorexia and ON risk.

#### 2.2.2. Orthorexia

The ORTO-15 is a 15-element tool for measuring the cognitive, rational, clinical, and emotional aspects of orthorexia developed in Italy [28]. Donini et al. [28] created the original nine items, using six of the ten items derived from the BOT. A four-point Likert scale was used to reflect how often a person exhibits attitudes towards healthy nutrition (4 = Always, 3 = Often, 2 = Sometimes, 1 = Never). Items that reflect an orthorexic tendency were scored as 1, and higher scores indicate more accurate eating habits. According to the original authors of the measure, a total score below 40 indicates orthorexia risk. One of the items in which the answer always indicates orthorexic tendency is, “At present, are you alone when having meals?” An example of a reverse item is “Is the taste of food more important than the quality when you evaluate food?” In the Polish adaptation of the scale using a complex multistep method, Brytek-Matera et al. [32] reported a reliability coefficient (Cronbach’s α) of 0.64; while Stochel et al. [31], in the other Polish adaptation, showed Cronbach’s α = 0.77.

#### 2.2.3. Eating Disorder

The Eating Attitudes Test (EAT-26) is an abbreviated 26-item version of the EAT-40, created by Garner et al. [46]. The first version of the scale, EAT-40, was originally developed to diagnose anorexia nervosa. EAT-26 is used in both clinical and non-clinical populations [47], as a screening method for various eating disorders, including anorexia nervosa [48], binge eating disorder [47], bulimia nervosa [49], and others. The questionnaire consists of 26 items, rated on a 6-point Likert scale (3 = Always, 2 = Usually, 1 = Often, 0 = Sometimes, 0 = Rarely, 0 = Never). Scores above 20 points indicate a real risk of ESDs. Higher results obtained by the addition of all 26 scores indicate higher risks of developing eating disorders. As the EAT-26 was found as having an unstable factorial structure (in particular, among non-clinical samples), ranging from three to seven factors in various studies [50], we decided to not consider the EAT-26 as a bi-factor hierarchical model with some number of subscales but, instead, used the total score as a sole indicator of disordered eating. The Polish version of the EAT-26 has been introduced in the study by Białokoz-Kalinowska et al. [51] and validated by Rogoza et al. [50], whereby the reliability coefficient was Cronbach’s α = 0.80.

#### 2.2.4. Disordered Eating Attitude

The Disordered Eating Attitude Scale (DEAS) is a tool developed by Alvarenga et al. [52] to measure disordered eating attitudes, which the authors define as abnormal beliefs, thoughts, feelings, behaviors, and relationships regarding food. The questionnaire consists of 25 items in five subscales: Relationship with food, Concerns about food and weight gain, Restrictive and compensatory practices, Feelings toward eating, and Idea of normal eating. The test has two parts: The first asks the participants to report which foods they consider healthy or unhealthy by checking one of the boxes “Eating this food often is healthy and necessary,” “Eating this food occasionally is healthy and necessary,” or “Not eating this food is healthy and necessary.” The last answer is connected with the highest mark. The individuals are also asked to answer questions about their eating practices and attitudes. The second part uses a five-point Likert scale, where higher scores indicate worse eating attitudes. The total internal consistency of the DEAS, as assessed by Cronbach’s α, was 0.67 [53]. The DEAS was translated into Polish for the present study, according to the guideline of Beaton et al. [54].

#### 2.2.5. Health Behavior

The Health Behavior Inventory (HBI) is a Polish scale developed by Juczyński [55]. The HBI consists of 24 items describing various health-related behaviors in four subscales (6 items included in each scale): Healthy Eating Habits (HEH; type of foods, well-balanced diet), Preventive Behavior (PB; health recommendations, health, and disease information), Positive Mental Attitude (PMA; psychological factors, such as avoiding too strong emotions, stress, anxiety, and depressive situations), and Healthy Practices (HP; sleep habits, recreation, and physical activity). Answers are rated on a 5-point Likert scale (1 = Almost never, 2 = Rarely, 3 = From time to time, 4 = Often, 5 = Nearly always). The total score of the HBI is a sum of all answers, ranging from 24 to 120 points, where a higher total HBI score indicates healthier behavior. The questionnaire’s reliability (in the Polish version) was satisfactory for the total HBI (Cronbach’s α =0.85) and acceptable for subscales, with Cronbach’s α ranging between 0.60 and 0.65 [55].

#### 2.2.6. Body Mass Index

Body mass index (BMI) was calculated by the investigators (BMI = weight/height^2^) from the weight (kg) and height (m) provided by participants in the questionnaire. According to the World Health Organization [56], particular BMI categories are interpreted as follows: BMI below 18.5 = underweight, BMI between 18.5 and 24.9 = average weight, BMI from 25.0 to 29.9 = overweight, and BMI above 30.0 = obese.

#### 2.2.7. Quality of Life

The World Health Organization Quality of Life (WHOQOL-Bref) was developed as an abbreviated version of the WHOQOL-100 quality of life assessment, designed for assessing the quality of life (QOL) in cross-cultural contexts [57,58]. The WHOQOL has been recommended as a useful tool for health professionals to assess and evaluate treatment efficacy. This self-reported questionnaire has been translated into several languages. According to the definition of the WHO, QOL may be understood as “individuals’ perceptions of their position in life in the context of the culture and value systems in which they live and in relation to their goals, expectations, standards, and concerns” [59]. The WHOQOL-Bref can assess one facet of the overall quality of life and general health (the sum of all 26 item scores, as well as QOL in four domains: Physical (PH), Psychological (PS), Social relationships (SR), and Environment (E). WHOQOL-Bref domain scores have shown good discriminant validity, content validity, internal consistency, and test-retest reliability [58]. Four types of 5-point Likert scale were applied to answer the particular items, regarding intensity, capacity, frequency, and evaluation of the respondent feelings during the last two weeks (e.g., how much, how completely, how often, how good, and how satisfied). A greater score indicates a higher QOL of the respondent. Skevington et al. [60] reported acceptable Cronbach’s α (above 0.70) for the total sample and physical health, psychological, environment, and social relationships domains, for which the Cronbach’s α values were 0.82, 0.81, 0.80, and 0.68, respectively. Similarly, Jaracz et al. [61] found acceptable internal reliability in the Polish population, with Cronbach’s alpha coefficients above 0.70 for all domains (except the social domain).

#### 2.2.8. Physical Health

The General Self-Rated Health (GSRH) is a measure of self-reported physical health developed by DeSalvo et al. [62]. The GSRH consists of only two items derived from the standard general health survey (SF-12V). In the first question, participants are asked to rate their health on a 5-point Likert scale (1 = Excellent, 2 = Very Good, 3 = Good, 4 = Fair, 5 = Poor). The second question asks participants to self-rate their health in comparison to other people of similar age. Higher scores in the GSRH indicate poorer health and are a good predictor of mortality [63]. Both questions were used in this research. A correlation has been found between both items (*r* = 0.74, *p* < 0.001). Research [62,63] has shown that the GSRH is a valid and reliable measure, with Cronbach’s α = 0.85 in a Polish sample [64].

#### 2.2.9. Anxiety

The Generalized Anxiety Disorder (GAD-7) is a brief 7-item clinical measure to screen for anxiety symptoms following DSM-IV criteria. The original version was developed by Spitzer et al. [65]. Participants answer the question “Over the last two weeks, how often have you been bothered by the following problems?” on a 4-point Likert scale (0 = Not at all, 1 = Several days, 2 = More than half the days, 3 = Nearly every day). Total scores range from 0 to 21, with a sum score of over 10 points indicating generalized anxiety disorder [65]. Spitzer et al. [65] reported the internal consistency to be very high (α = 0.92). In the Polish study [65], the reliability coefficient of GAD-7 was also high (Cronbach’s α = 0.92).

#### 2.2.10. Depression

The Patient Health Questionnaire (PHQ-9) is a 9-item brief depression severity measure developed as a screening test according to the DSM-IV diagnostic criteria [66,67]. Individuals answer each statement on a 4-point Likert scale (0 = Not at all, 1 = Several days, 2 = More than half the days, 3 = Nearly every day), indicating how often (over the last 2 weeks) they have been bothered by particular depression symptoms. A PHQ-9 score above 10 indicates major depressive disorder [66]. The internal reliability of the PHQ-9 has been shown to be good (Cronbach’s α = 0.89). In a Polish validation study [68], Cronbach’s α of the PHQ-9 was 0.88.

#### 2.2.11. Obsessive-Compulsive Disorder

The Obsessive-Compulsive Inventory-Revised (OCI-R) was developed by Foa et al. [69], as a short version of the self-reported Obsessive-Compulsive Inventory (OCI), assessing symptoms of obsessive-compulsive disorder (OCD). This revised scale consists of 18 items, in which participants rated on a 5-point Likert scale (0 = Not at all, 1 = A little, 2 = Moderately, 3 = A lot, 4 = Extremely), how much the given experience distressed or bothered them during the past month. The total score is the sum of all items, where higher scores (i.e., above 21) indicate more severe OCD. The tool also consists of six subscales representing subtypes of OCD symptoms: Washing (“I find it difficult to touch an object when I know strangers or certain people have touched it”), checking (“I check things more often than necessary”), ordering (“I get upset if objects are not arranged properly”), obsessing (“I find it difficult to control my own thoughts”), hoarding (“I have saved up so many things that they get in the way”), and mental neutralizing (“I feel compelled to count while I am doing things”) [70]. The Polish version of the OCI-R was translated from English by Jeśka [71]. As measured by Cronbach’s α, its reliability is very good: 0.90 for the total OCI-R score and ranging between 0.83 and 0.90 in the particular subscales [70].

### 2.3. Procedure

#### 2.3.1. Study Design

A cross-sectional pilot study was conducted in a sample of 24 psychology students in order to assess whether the items were grammatically correct, clearly formulated, and understandable. According to the collected opinions about the test, minor corrections were introduced. The final form of the 40-item Test of Orthorexia Nervosa (TON-40) was used to conduct a preliminary evaluation, and its psychometric properties, discriminant accuracy, and validity were assessed.

A cross-sectional study was conducted using two forms of the test—an online survey (*n* = 339) and a paper-and-pencil questionnaire (*n* = 428)—both recruiting a convenience sample from the general population. The link to the online Google form was disseminated, by e-mail (using a snowball technique), to acquaintances, friends, and family members. Concurrently, the link to the online survey form was distributed through social media, such as personal Facebook websites and Facebook groups related to a healthy lifestyle and healthy food. Inclusion criteria were being aged 18 years and older.

All surveys included the TON-40 and questions related to demographic characteristics, eating habits, reasons for sticking to a restrictive diet, and allergies or chronic diseases being a cause for dietary restrictions. Some other standardized questionnaires were also included in the surveys in order to measure ON (ORTO-15), attitude to eating (DEAS), EATs (EAT-26), health-related behavior (HBI), physical health (GRSH), and several mental health dimensions, such as quality of life (WHOQOL-Bref), anxiety (GAD-7), depression (PHQ-9), and OCD (OCI-R). Although all 767 participants responded to demographic and TON-40 questions, various other questionnaires were added to particular surveys not to exceed 100 items in one survey (see Table S2).

The paper-and-pencil questionnaires were disseminated, using a snowball technique, among healthcare professionals in one hospital, soldiers in one military unit, and students at three universities. University students participated in the study during classes at the university, with the permission of lecturers. One sample of psychology students (*n* = 133) participated in the prospective study twice, with four weeks of break-out between test and retest. The mean scores were used to replace missing values (less than 5%).

Written informed consent was obtained from all participants before enrollment. Information about the study and informed consent was presented on the first page of the survey, and participants were required to click an acceptance box if they agreed to participate. Respondents participated anonymously and voluntarily in the research. All procedures followed the responsible committee’s ethical standards on human experimentation (institutional and national) and the Helsinki Declaration of 1975 (revised in 2000). Informed consent was obtained from all participants for being included in the study.

#### 2.3.2. Statistical Analysis

**Figure 1:**
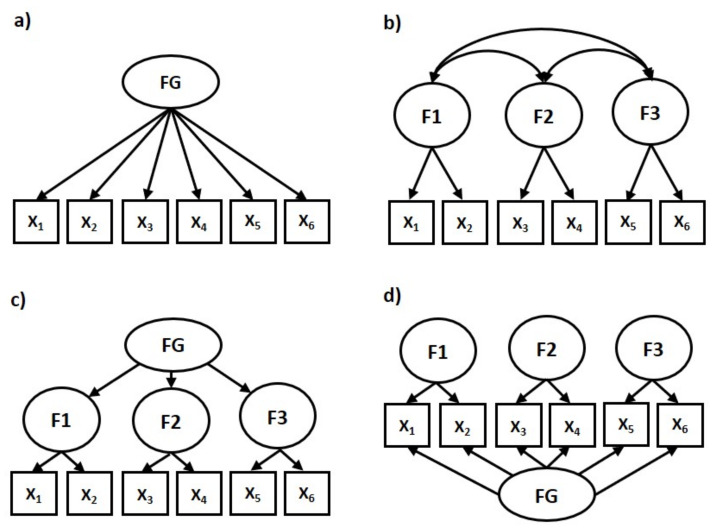
Hypothetical models of factor analysis: (**a**) One-factor; (**b**) Three-factor; (**c**) Second-order factor; (**d**) Bi-factor. FG = general factor, X_1_–X_6_ = Items; F1–F3 = factors. Latent (factor) variables are represents by ovals, while observed (measured) variables are represented by rectangles.

Psychometric properties of the TON were examined, in regards to the range of scores, mean (*M*), 95% confidence interval (*CI*), standard deviation (*SD*), standard error (*SE*), median (*Mdn*.), kurtosis, skewness, Kolmogorov–Smirnov *d* statistic for normality of distribution, item-total correlations, and Cronbach’s α reliability coefficient. Furthermore, *M*, *SD*, and Cronbach’s α were tested for the other variables measured on the scales of ORTO-15, DEAS, EAT-26, GRSH, HBI, WHOQOL-Bref, GAD-7, PHQ-9, and OCI. Pearson’s correlation coefficient *r* was used to test the relationship of TON with other scales and between particular items within the TON. Group differences were examined using Student’s *t*-test, whereas frequency in particular groups was compared using contingency tables and Pearson’s χ^2^ test. The following statistical tests were performed to reduce items and to check the TON structure: reliability analysis, exploratory factor analysis (EFA), and second-order confirmatory factor analysis (CFA). The total sample (*N* = 767) was randomly divided into three samples for EFA and CFA analysis: Sample 1 (*n* = 256), Sample 2 (*n* = 256) and Sample 3 (*n* = 255). The inter-correlations between items of the TON-40, the Kaiser–Meyer–Olkin (KMO), and Bartlett’s test of sphericity were initially performed to explore the data’s properties. Results indicated that a hierarchical factor model (also called a higher-order factor model) is the most appropriate to test whether ON has a multi-level structure. For this purpose, the Statistica software was used, which first identifies clusters of items and rotates axes through those clusters. Next, the correlations between those (oblique) factors are computed. That correlation matrix of oblique factors is further factor-analyzed to yield a set of orthogonal factors that divide the variability in the items into that due to shared or common variance (secondary factors), and unique variance due to the clusters of similar variables (items) in the analysis (primary factors). As a result, a general (secondary) factor can be identified at the higher level (that likely affects all items), and some number of primary unique factors at the lower level of the bi-factor structure (see Figure 1c for more details). A CFA was performed to examine whether the data fit the measurement model derived from the EFA solution. We explored four hypothetical models: (a) One-factor; (b) Three-factor; (c) Second-order factor; (d) Bi-factor (Figure 1). The ADANCO software [72] was used to estimate the model with partial least squares path modeling (PLS-PM). STATISTICA ver. 13.5 [73], IBM SPSS ver. 25 [74], and AMOS ver. 20 statistical software were used to calculate the results in this study.

## 3. Results

### 3.1. Internal Item Qualities

Initially, the internal item qualities analysis was performed in the total sample (*N* = 767) to examine properties of items that can be assessed in reference to other items on the scale or in reference to the scale’s summated scores. The item-total correlation is frequently used for reducing the length of self-report questionnaires. The assessment of scale reliability is based on the correlations between the individual items or measurements that make up the scale relative to the variances of the items. An index of reliability is the proportion of true score variability that is captured across subjects or respondents relative to the total observed variability. All 40 item characteristics showed good properties, distinguishing the respondents appropriately (Table S3). The item means (*M* = 91.06, ranging from 87.48 to 89.41 if the item was deleted) and standard deviations (*SD* = 25.73, ranging between 24.75 and 25.35 if the item was deleted) showed no extreme values or nearly zero variance (*S*^2^ ranging between 618.04 and 630.00 if the item was deleted). Furthermore, Cronbach’s α was 0.93 for the total score and for each item (ranging from 0.929 to 0.934 if the item was deleted), indicating high internal consistency. The average inter-item correlation was *r* = 0.28, whereas the correlation coefficient between individual items and the total score ranged between 0.24 and 0.73. As the reliability indices showed very good properties, this strategy was not useful in reducing the TON-40 scale. Factor analysis was then performed for scale reduction.

### 3.2. Test-Retest Reliability

Test-retest reliability (Table S4) was assessed in a convenience sample of 150 Psychology students (undergraduates of second-year *n* = 68 and third-year *n* = 82). The test and retest were conducted during the classes at the University, with a duration time of 4 weeks. As 21 students were not present at the first and/or second test, and as three of the students refused participation in the study, the final number of students participating in the test and retest was *n* = 126. The test-retest correlation coefficients, including Cohen’s Kappa *K*, Pearson’s *r*, and Spearman’s *r*, are shown in Table S2. Cohen’s Kappa coefficient was used to estimate interrater reliability, which indicates the extent of agreement between frequencies of two sets of data collected on two different occasions. The Cohen’s Kappa coefficient ranged from 0.21 to 0.48. The internal consistency of the first test of the TON-40 were as follows: Cronbach’s α = 0.90, mean correlation between items = 0.20, Spearman–Brown’s spilt-half reliability = 0.92, Guttman’s spilt-half reliability = 0.92, and the correlation between first and second half = 0.84. In comparison, the reliability coefficients for the re-test were Cronbach’s α = 0.94, mean correlation between items = 0.29, Spearman–Brown’s spilt-half reliability = 0.96, Gutman’s spilt-half reliability = 0.96, and the correlation between first and second half = 0.91.

### 3.3. Investigating the Factor Structure of the TON-40 Using Exploratory Factor Analysis

In the first step of factor analysis, EFA 1 was performed in Sample 1 and EFA 2 in Sample 2, for comparison. We expected one overall higher-level factor to be split into several numbers of lower-level factors (Figure 1c). Hierarchical factor analysis has been found to be suitable for some previous ON tools, such as EHQ and ONS [1].

First, the data were tested for suitability for factor analysis. The Pearson’s correlation analysis was performed for all 40 items in Sample 1 and Sample 2, separately. Inter-correlations were significant for most of 40 items, showing weak, moderate, or strong relationships in both Sample 1 and Sample 2 (Table S5). The Kaiser–Meyer–Olkin (KMO) test was 0.677 for Sample 1, and 0.745 for sample 2, indicating both samplings are adequate. The Bartlett’s test of sphericity yielded χ^2^(780) = 1696.214 (*p* < 0.001) in Sample 1, and χ^2^(780) = 2020.538 (*p* < 0.001) in sample 2, suggesting a factor analysis may be useful with the data in both groups. Initial analysis of the correlation matrix, KMO, and Bartlett’s test, indicates that the hierarchical EFA model is the best solution. The hierarchical second-order factor model was examined with the principal axis extraction and varimax rotation method. The Scree test and Kaiser’s stopping rule (eigenvalue ≥ 1) were used to determine the optimal number of components; both methods indicated a three-factor solution in both samples [75,76]. Three primary factors and one secondary factor were loaded in both samples.

The EFA 1 (Sample 1) showed one higher-order general factor (FG) and three following primary factors for the TON-40: Factor 1 (eigenvalue 11.31, explaining 28.28% of the total variance), Factor 2 (eigenvalue 4.09, explaining 10.23% of the total variance), and Factor 3 (eigenvalue 1.72, explaining 4.31% of the total variance). Three primary factors were found in EFA 2 (Sample 2), besides second-order factor: Factor 1 (eigenvalue 12.47, explaining 31.16% of the total variance), Factor 2 (eigenvalue 4.18, explaining 10.46% of the total variance), and Factor 3 (eigenvalue 1.77, explaining 4.43% of the total variance).

The following two criteria were used to reduce the items: (1) if loading was lower than 0.40 the item was deleted; (2) if the items had loadings on more than one factor at minimum 0.30 level, a cross-loading was assumed and such item was removed from further analysis. Factor loadings ranging between 0.30 and 0.40 are most frequently considered to meet the minimal level for interpretation of structure [77]. Seventeen items were discarded in Sample 1, while 18 items in Sample 2 (see Table S6). The TON-23 (EFA 1 conducted in Sample 1) included 7 items in Factor 1 (items 2, 11, 19, 25, 26, 27, 38), 9 items in Factor 2 (1, 3, 4, 5, 8, 12, 13, 17, 34), and 7 items in Factor 3 (15, 22, 28, 29, 32, 36, 37). The TON-22 (EFA 2 performed in Sample 2) comprised 8 items in Factor 1 (1, 3, 4, 5, 8, 10, 16, 17), 8 items in Factor 2 (2, 6, 11, 19, 27, 38, 39), and 6 items in factor 3 (22, 28, 29, 32, 36, 37). The comparison of both solutions leads to the conclusion that some items were repeated in both EFAs (in Sample 1 and Sample 2), while some items were contained only in TON-22, but not in TON-23, and conversely (Table S6). All single items shown in only one EFA result were rejected in the third step of item reduction, while all items presented in both Sample 1 and Sample 2 were included in the TON-17 final scale.

The final step in the exploration of EFAs was to test if TON-17 would show a stable structure by repeating the factor solution in Sample 3. The initial correlation analysis (Table S7), as well as the Kaiser–Meyer–Olkin (KMO) test (0.886), and Bartlett’s test of sphericity showed adequate properties for factor analysis, χ^2^(780) = 4711.470, *p* < 0.001. Similar to the previous analysis, the hierarchical second-order EFA 3 was conducted in Sample 3. The EFA 3 fully replicated the three-factor solution in a lower-order, including items 1, 3, 4, 5, 8, and 17 to Factor 1; items 2, 11, 19, 27, and 38 to Factor 2; and items 22, 28, 29, 32, 36, and 37 to Factor 3 (Table S6).

### 3.4. Examining the Factor Structure of the TON-40 Using Confirmatory Factor Analysis

The confirmatory factor analysis (CFA) with maximum likelihood (ML) estimation procedure was conducted to examine how well the models found previously in the EFA reproduced the data. The following fit indices were used to test the factor models: the proportion between ML χ^2^ and its degree of freedom *df*, the standardized root mean square residual (SRMR), root mean square error of approximation (RMSEA), comparative fit index (CFI), normed fit index (NFI), Akaike information criterion (AIC), and Bayesian information criterion (BIC). Four parallel structural models were examined for the TON-22, TON-23, and TON-17: one-factor, three-factor, second-order factor, and bi-factor (Figure 1a–d, respectively). The CFA 1 was cross-match performed in Sample 1 to verify the EFA 2 result (TON-22), while CFA 2 was conducted in Sample 2 to check EFA 1 solution (TON-23). Further, the CFA 3 was carried out in Sample 3 to test the stability of the TON-17 structure, deriving comparatively from Sample 1 and Sample 2. As shown in Table 2, acceptable fit indices were found for the bi-factor model of TON-22, TON-23, and TON17 and a three-factor model of TON-23 [78,79,80,81,82,83]. However, the best-fit indices were found for the bi-factor model of the TON-17. Therefore this model was selected for further statistical analysis.

### 3.5. Confirming the Factor Structure of the TON-17 Using Composite Construct Analysis

**Figure 2:**
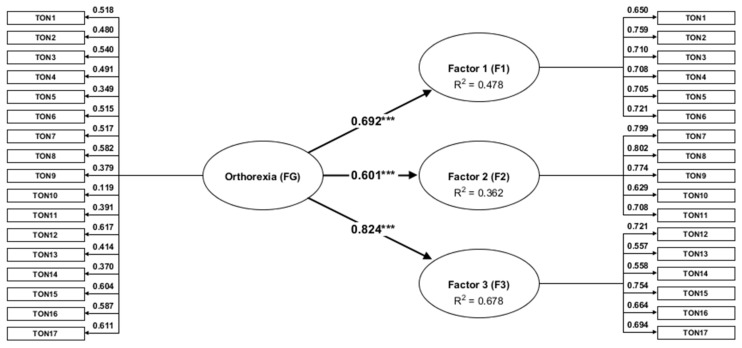
Orthorexia bi-factor reflective model. *** *p* < 0.001.

The structural analysis’s last step was to confirm the TON-17 bi-factor solution using the composite construct analysis (CCA). Partial least squares path modeling (PLS-PM) is a statistical approach for modeling complex multivariable relationships (structural equation models) among observed and latent variables. The composite modeling is a modern approach that explicitly considers the proxy nature of construct measures, which may produce more realistic assessment of factor structure than CFA [84,85,86]. The PLS-PM was performed, with one general factor higher-order (FG) and three lower-order factors (Factor 1, Factor 2, and Factor 3). The path diagram for the reflective model of the PLS-PM is shown in Figure 2.

Firstly, the construct’s reliability was examined in the composite measurement models, calculating Cronbach’s α and composite reliability (CR). The values of both internal consistency reliability indices (α and CR) should exceed 0.70 to confirm good properties. As it is shown in Table 3, both Cronbach α and CR indicate sufficient reliability for all reflective constructs Factor 1 (F1), Factor 2 (F2), Factor 3 (F3), and General Factor (FG). It is important to note that the CR (weighted) is considered more accurate than the Cronbach α (unweighted) reliability coefficient. Furthermore, the values ranging between 0.70 and 0.90 indicated that individual items within three factors measure various concepts and are not redundant. Also, Dijkstra–Henseler’s ρ_A_ was 0.80, 0.83, 0.76, and 0.81, for factors F1, F2, F3, and FG, respectively. If a Dijkstra–Henseler’s ρ_A_ is larger than 0.707, it suggests that the latent variables can explain more than 50% of the construct scores variance. As such, the reliability of multi-item constructs was confirmed.

The average variance extracted (AVE) analysis was performed to measure the convergent validity of the scales. The AVE measures the average variance shared between the construct and its individual indicators. Of all the factors, F1 and F2 meet the AVE > 0.50 criterion, while F3 and FG do not exceed the threshold for acceptable convergent validity. The variance explained by the F3, and FG latent variable is less than the measurement error. However, according to Fornell and Larcker [87] the AVE < 0.5 can be accepted if the CR is > 0.6, because the convergent validity of the construct is still adequate. Convergent validity is the extent to which the indicators belonging to one latent variable actually measure the same construct. However, it seems reasonable that the higher-order latent variable FG cannot measure a single construct if it includes three factors. Also, F3 seems related to various mental disorders, which may lead to a less unequivocal solution. On the other hand, the F3 may simply not comprise all the disordered symptoms of orthorexia. Therefore it explains a small percentage of variance. Indicator reliability, assessing through the factor loading estimates, ranged from 0.56 to 0.80 for the primary factors and from 0.12 to 0.62 for the second-order factor. The loadings values exceeding 0.707 indicate that the corresponding latent variable can explain more than 50% of the variance in a single indicator. However, as long as the construct validity and reliability criteria are met, lower AVE values could not be considered as problematic.

Discriminant validity means that two latent variables that are intended to represent two different concepts are statistically sufficiently different. Discriminant validity is demonstrated when the shared variance within a construct (AVE) is greater than the shared variance between the constructs. The HTMT lower than 0.85 indicates sufficient discriminant validity throughout the three factors. The highest HTMT value between the F1, F2, and F3 factors was 0.41. In contrast, the HTMT value between F3 and FG equals 1.02, which suggests that these two factors do not differ significantly from each other.

Examining multicollinearity showed that the VIF values for the TON-17 items as indicators of the composite models range from 1.33 to 2.09 for all three factors (F1, F2, F3), as well as for the general factor (FG). High multicollinearity can lead to insignificant estimates and unexpected signs of the weights. The present result suggests that multicollinearity is not a problem in the data since the VIF > 5 is regarded as problematic.

The estimated model’s overall fit should also examine the path coefficient estimates, the effect sizes (*f*^2^), and the coefficient of determination *R*^2^ in explanatory research. The path coefficient estimates for the relationships between the three lower-order and one second-order latent variables range from 0.601 to 0.824, with a significance level *p* < 0.001 (Figure 2). The effect size is a measure of the magnitude of an effect that is independent of sample size. The Cohen’s *f*^2^ equals 0.92 for FG→F1, 0.57 for FG→F2, and 2.11 for FG→F3 relationship, which means a large effect size. The regression analysis also includes *R*^2^ for assessing goodness of fit. As it is shown in Table 3, the *R*^2^ values for F1, F2, and F3 are 0.48, 0.31, and 0.68, respectively. Items 1–6 explain 48% of the F1 variance (control of food), items 7–11 explain 31% of the F2 variance (fixation on health and healthy diet), while items 12–17 explain 68% of the F3 variance (disorder symptoms). 

### 3.6. Descriptive Statistics of the TON-17 Scales

The scores from items 1-17 of the TON-17 were summed up to the total score that represents the FG factor, scores of items 1–6 to the F1 scale, scores of items 7–11 to the F2 scale, and scores of items 12–17 to the F3 scale. The descriptive statistics of the TON-17 are presented for Sample 3 (*n* = 255) in Table 4. Although the Kolmogorov–Smirnov statistics indicate that the TON-17 scales do not meet the assumption of normality (*p* < 0.01), the skewness and kurtosis analysis suggest that the scales presented good psychometric properties (ranging from −2 to 2), except Factor 3. 

Analysis of reliability of the FG of TON-17 showed good internal consistency (Table S8). The Cronbach’s α ranged from 0.75 to 0.79 for particular items, if the item deleted. Item-total correlations ranged between 0.22 and 0.50 for the individual 17 items. Internal consistency coefficients were also satisfactory for the TON-17 subscales (Cronbach’s α ranging between 0.73 and 0.81, for Sample 3). However, in the total sample, Cronbach’s alphas increased to 0.82, 0.79, 0.80, and 0. 81, for F1, F2, F3, and FG, respectively. Pearson’s *r* coefficient in Sample 3 (*n* = 255) indicated moderately strong correlations between the total scores and subscales of the TON-17 (Table 4). Factor F3 is weakly correlated with F1 and F2, while factors F1 and F2 are uncorrelated.

The 95th percentile estimation was performed for the FG of the TON-17, to examine prevalence of orthorexia risk in the total sample (*N* = 767). In epidemiological studies, percentiles are recommended for reference interval estimation of continuous data [88]. The 95th percentile was calculated using the SPSS software, with Bootstrapping method of 1000 samplings. The method of percentile estimation calculates a rank *p* (*n* + 1) with *p* representing the centile (divided by 100) and *n* the sample size. The cut-off score of 61 was found for the total TON-17, Bootstrap estimation 61.6, *SE* = 1.18, 95% *CI* (59.00–63.60). Of the 767 participants, 42 participants (5.48%) presented total scores equal or greater than 61.

### 3.7. Construct Validity of the TON-17

The mean TON-17 scores were compared in groups of healthy people with those to whom medical conditions or diseases determined a special diet in their every-day activity. Gender differences were also examined in the total sample. Intergroup differences are shown in Table 5. People with medical reasons to follow a restrictive diet scored significantly higher than the healthy sample in Factor 1 of the TON-17 (which characterizes control of food quality), but with a small effect size. Women scored higher than men in the total TON-17 (FG) and Factor 2 with small effect size, while in Factor 1 with medium effect size.

The construct validity was examined using Pearson’s correlation between the TON-17, and such variables as ORTO-15, EAT-26, DEAS, HBI, GRSH, Brief WHOQOL, GAD-7, PHQ-9, and OCI-R. The correlations of the TON-17 scales with other variables are presented in Table 6. The TON-17 was significantly but weakly correlated with the ORTO-15 and EAT-26. The disordered eating attitude was also positively, but weakly, associated with TON-17 and its F1 and F3 scales. Restrictive practices and feeling toward eating (of the DEAS) were negatively and weakly associated with the F2. Moderate correlations were found between the total TON-17, F1, and F2 as an orthorexia dimension and both scales of healthy behavior HEH and the total HBI. Also, weak correlations were noticed of FG, F1, and F2, with PB, PMA, and HP of the HBI. The BMI was significantly but extremely weakly related to FG and F3. Quality of life scales was positively but weakly associated with F2, while physical health was negatively and weakly related with F2. Obsessive-compulsive disorder correlated weakly or moderately with all scales of the TON-17 (except for insignificant relationship of F1 with obsessing and hoarding). Anxiety and depression were weakly associated with F1, F2, and FG.

## 4. Discussion

### 4.1. Structure of the TON

The present study showed the development and validation of the Test of Orthorexia Nervosa TON). The initial version of the questionnaire (TON-40) was reduced to 17 items (TON-17) as a result of the structural analysis. The final version of the TON-17 includes three subscales factors (Control of food quality, Fixation of health and healthy diet, and Disorder symptoms) in a hierarchical, bi-factor structure. The TON-17 seems to be a promising tool for assessing the risk of orthorexia nervosa. According to the recommendations of Valente et al. [41], the current ON measurement was developed based on qualitative methods (e.g., interviews) and state-of-the-art ON reconceptualization. Furthermore, statistical techniques such as EFA, CFA, and CCA supported the structural construction of the TON-17.

The EFA extracted a higher-order factor structure, with one secondary factor (general ON) and three lower-level primary factors. The F1 is related to the need for control over food quality and diet preoccupation. The F2 seems to refer to an excessive fixation on health, healthy behavior, and health-related quality of life. A healthy diet may be one of many other practices of a healthy lifestyle to achieve longevity. The F3 scale demonstrates a stronger relationship with mental disorder symptoms, such as OCD, anxiety, and depression, compared to the other scales. Furthermore, the CCA showed that F3 does not distinguish substantially from FG, indicating that Factor 3 represents the strongest association with orthorexia.

Using EFA, CFA, and CCA, the present study found evidence that orthorexia may demonstrate a bi-factor structure, with one higher-order general factor as a global orthorexia dimension and three lower-order factors. A three-factor structure was previously identified in orthorexia research using the DOS, EHQ, and ORTO-15 [1]. However, the other ON tools indicated different factor structures, ranging from one to 10 factors. Therefore, more research is necessary to confirm the three-factor structure as more appropriate to ON’s nature compared to other structural solutions. 

The prevalence of ON according to TON-17 (95th percentile) was 5.5%. A cut-off point of 61 is recommended for the assessment of the risk of ON. However, further studies are needed to verify the accuracy of the threshold in terms of sensitivity and specificity. The prevalence of ON found in this study is consistent with recent studies using DOS which showed the prevalence rates between 2.3% and 7.8% in various studies [23,24,25,26,27].

The scales F1 and F2 appear to be associated with healthy orthorexia, while F3 to unhealthy orthorexia. Factor 2 is based on hedonistic motivation to be healthy and fit and on a positive attitude towards a healthy lifestyle, while Factor 1 is based on the need to control life through healthy eating and excessive worry about health. Both F1 and F2 are focusing on health. By contrast, F3 has lost its balance in areas such as emotions, thinking, and behavior. Fixation on health leads to adverse symptoms of mental and physical health.

The present study is consistent to some extent with the previous study, which found that orthorexia consists of two dimensions: healthy and unhealthy [35,43,89,90]. The content of the TOS resembles the TON, to some extent [89]. However, in the TOS, healthy and unhealthy orthorexic tendencies are included in two separate scales. In the TON, otherwise, all three factors indicate that healthy and pathological inclinations are only a matter of intensity since one general factor was justified in this study. Factor 2 of the TON (excessive fixation on health and healthy diet) positively correlates with life quality. People who eat healthily may initially feel great. This relative sense of satisfaction with life can exclude other types of food from the diet that do not appear to be healthy enough. Such an illusory feeling of control over health may be dangerous if the restrictive behavior is perpetuated and expanded. As with all disorders, the line between healthy and unhealthy eating can be fluid and dependent on many factors. Further research should be aimed at identifying the stages of developing ON, the factors contributing to pathological ON, and establishing clear criteria for determining healthy and unhealthy levels of ON.

From an evolutionary perspective, Bóna et al. [91] found that some of the orthorexic traits meet the conditions of becoming adaptive drivers of human behavior, whereas others show non-adaptive health behavior tendencies. Indeed, orthorexia may be conceptualized as complex eating behaviors that include pathological and non-pathological health dimensions [37,89]. Strahler [43] also found two healthy and unhealthy orthorexia dimensions by using the Teruel Orthorexia Scale (TOS). More research should be performed in order to confirm the dual nature of orthorexia (healthy and unhealthy).

All three scales of the TON-17 showed a weak and positive correlation with EDs, which seems to confirm the convergent validation of the TON-17. The positive correlation between ON and EDs has been confirmed in some previous studies [6,13,38,41]. Moreover, Zickgraf et al. [42] indicated that ON symptoms are more strongly correlated with anorexia and bulimia than ARFID. Research has indicated that individuals with ON scored significantly higher in an eating disorder test (the EAT-26) than a non-clinical control group [38,39]. On the other hand, a recent study [41] found a high prevalence of ON in patients with anorexia and bulimia. Therefore, ON is usually categorized within the spectrum of disordered eating.

The relationships between ON and EDs and/or OCD have been extensively reviewed previously [6,11,13]. Koven and Abry [11] examined the symptoms of comorbid psychopathology of ON and other disorders, including anorexia nervosa, obsessive-compulsive disorder (OCD), obsessive-compulsive personality disorder (OCPD), somatic symptom disorder, anxiety disorder, and psychotic spectrum disorders. They found that shared traits between ON and OCD include intrusive thoughts, ritualized food preparation, and focus on contamination; shared traits between ON and AN were limited insight, guilt over food transgressions, and ego-syntonic thoughts; while shared traits linking ON, AN, and OCD were perfectionism, cognitive rigidity, trait anxiety, impaired working memory, impaired functioning, and poor external monitoring.

Addictive behavior has been found in the present research for the first time, to the best of our knowledge. However, several previously described traits that characterize ON [6,11,13,17,18,33,41,45,92,93] seem similar to symptoms of addictive behavior, including (1) excessive focus on healthy food and associated behaviors, such as preparing and shopping, that takes up most of the individual’s time; (2) feelings of frustration, irritation, and anger if healthy food cannot be accessed, or a sense of guilt and shame after transgressing dietary rules; (3) loss of interests and hobbies that were previously important; (4) impairment of social, academic, or vocational functioning caused by restrictive eating; (5) decreased physical health caused by the restricted diet, and not changing behaviors, despite harm to self or others. The other shared traits of ON and addictive behavior are perfectionism, impaired cognitive control and inhibitory control over behaviors related to healthy practices, and a tendency to dominate a stimulus-driven behavior associating with rewarding and reinforcing healthy food. Further research should verify the relationship between ON and addictive behavior and replicate the present three-factor structure.

The psychometric properties of the scales and subscales are good. Individual scales of the TON-17 were positively correlated with weak or moderate strength. These correlations justify the hierarchical bi-factor structure of the TON-17. The reliability measures (Cronbach’s α, CR, and Dijkstra–Henseler’s ρ_A_) confirm that the TON-17 is a reliable orthorexia tool. Furthermore, the test-retest replicability demonstrated acceptable correlations after four months. Internal consistency (Cronbach’s α and spilt-half Spearman–Brown coefficient) even increased in the retest. However, the dynamics of ON changes is unknown, so the four-week period between testing and retesting may include some changes related to ON development. The other CCA indices also confirm that the three-factor model with one second-order general factor represents the appropriate structure. The discriminant analysis showed that F1 and F2 demonstrate distinguishable concepts, while FG and F3 are undifferentiated. Instead, these results confirm that F3 is the strongest measure of orthorexic risk tendencies and that all three factors are associated with ON since F1, F2, F3, and FG are correlated with each other and FG has sufficient internal consistency. It should be noted that item-reduction strategies may increase the risk of the construction measures being too narrow and loss of reliability. The TON-17 is a relatively short tool, with minimal loading of the item to each scale and without excessive costs to reduce internal consistency. Furthermore, the bi-factor model was fully reproduced in Sample 3, proving a stable construct. TON-17 covers the three aspects of orthorexia, focusing on a healthy lifestyle as well as unhealthy symptoms, which is in line with recent findings by using the other ON tools [35,43,90].

### 4.2. Construct Validity of the TON

In the present study, we showed the ambiguous relationship of gender with ON. Total scores of the TON-17 and its factors 1 and 2 indicate that women scored significantly higher than men, while no gender difference was found in factor 3. Similar unequivocal results have also been found in previous studies [6,13,36]. Therefore, more research is necessary in the future in order to resolve the question of gender differences in ON.

A very weak and positive association was found between body mass index (BMI) and ON (i.e., in the FG and F3 scales of the TON-17), which seems consistent with some previous studies [6,13]. A recent review [13] has shown that gender is unrelated to ON, but ambiguous results have also been found for the associations between ON and age, socioeconomic status, and BMI. Varga et al. [6] have reported that most of the reviewed studies had found no association between ON and BMI. However, higher BMI scores were positively related to more healthy dietary attempts, especially in combination with other variables, including medical reasons, dieting, and healthy nutrition.

Consistent with our assumption, positive correlations were found between TON-17 and the other measure of ON (the ORTO-15), disordered eating (the EAT-26), and obsessive-compulsive disorder (the OCI-R). However, the correlation between TON-17 and ORTO-15 is weak. ORTO-15 has been criticized as a weak measure of pathological dietary tendencies, poor validity and low reliability. Further research should verify the compatibility od TON-17 with more valuable ON tools such as EHQ-R, TOS and DOS. A positive correlation between ON and EDs was expected due to previous research and the classification of ON within the eating disorders spectrum [1,9,11,13,38,39,40,41,42]. However, Barthels et al. [37] showed that people with restrained eating patterns focus more on quantity than food quality. Therefore, orthorexia cannot be considered equivalent to bulimia or anorexia nervosa.

Other relationships of the TON-17 with disordered eating attitudes and special diets, such as meatless, pure food, or color diets, can also confirm the accurate properties of the TON-17 [3,12,13,14]. However, it is essential to note that various feeding and eating disorders demonstrate distinctive features and developmental and cross-cultural differences, as has been evidenced in a review study [89]. Thus, cross-cultural research should be conducted using the TON-17 to examine ON’s relationships with both disordered and healthy eating behaviors.

The obtained correlations of ON with indices of mental health disorders were consistent with the previous studies using other orthorexia measurement tools, which may confirm the convergent validity of TON-17. Positive associations between ON and obsessive-compulsive disorder, anxiety, and depression have been found previously [11,15,43]. Some shared traits of ON and OCD determine a strong correlation between the two disorders [11]. Furthermore, anxiety, as a trait, is strongly related to OCD. Moreover, some studies have indicated that ON is related to a predominance of negative emotions [6] and difficulties in identifying and regulating emotions [93]. This characteristic of the emotional sphere of life may predispose people with ON to developing depression.

For the first time, the present study examined the relationship between ON and various dimensions of healthy behaviors. People with ON seem to demonstrate a wide range of healthy control behaviors, such as preventive behavior, positive mental attitude, healthy practices, and healthy eating habits. Almost all scales of the TON-17 were moderately or weakly correlated with healthy behaviors. This fact may explain why people with a higher risk of ON tend to overestimate their health (negative correlation of the GSRH with Factor 2 of the TON-17, which mean excessive fixation on health), and quality of life in such domains as physical, psychological, and environmental (positive association of WHOQOL with Factor 2 of the TON-17).

Previous studies have found a negative relationship of ON with self-reported physical health [6,9] and quality of life [43]. However, Strahler [43] has shown that healthy orthorexia was positively associated with wellbeing. Furthermore, Strahler [43] also showed that ON is associated with pathological consequences, while healthy orthorexia may buffer these consequences. Poorer mental health was associated with ON among women, whereas better mental health was related to healthy orthorexia in men. Future research should aim to replicate the gender-specific mediation analysis [43] using the TON-17 subscales.

Varga et al. [6] have reported that a somatic illness is usually the first step to introducing a diet, leading to an excessive interest in healthy nutrition and ON. It seems possible that people who follow a healthy diet feel a higher sense of health control and can better assess their health, with respect to the earlier perception of the health condition before they started following the diet. On the other hand, the present study does not indicate intergroup differences in ON between individuals with medical conditions requiring a special diet and those without such problems. The only statistically significant (but weak) difference was found in F1 of TON-17 (obsessive control of food quality). The sample with a disease requiring a special diet presented higher ON scores than the group without medical problems. More research is needed to explain the obtained results.

A recent review [1] has found that most of the identified ON measurement questionnaires failed in robust psychometric properties and validation. The construct validity of TON-17 was investigated, herein, for 13 variables, including other ON tool (ORTO-15), eating disorders (the EAT-26 and DEAS), healthy behavior (the HBI), quality of life (the Brief WHOQOL), physical health (the GRSH), anxiety (the GAD-7), depression (the PHQ-9), obsessive-compulsive disorder (the OCIR), and demographic variables such as gender, BMI, vegetarian dieting, and the medical reason for a restrictive diet.

Although there are several measures of ON, including BISQ [94], B-ORA [95], BOT [33], DOS [96], EHQ [97,98], ONS [99], ORTO-15 [17,28], the Puerto Rican ON Tool [100], and TOS [88], each seems to assess some narrow aspect of ON [1]. For example, the BISQ aim was the early detection of various eating disorder behaviors. B-ORA addresses the cognitive, emotional, and behavioral current problems related to ON. The BOT was designed to identify potential problems with eating habits. DOS is a short scale of 10 items that measures potentially pathological healthy diet. EHQ and EHQ-R assess pathological persistence in the healthy food. ONS measures certain ON-related behavioral trends (i.e., superiority, inferior social comparison, stiffness, cleanliness, social avoidance, identity, eating disorders as meaning, loss of control, preoccupation, eating to cope, nutritional deficiencies, and relationship problems). ORTO-15 was developed to assess the cognitive-rational, clinical and emotional aspects of ON. The Puerto Rican ON tool was developed as an inclusive tool that is applicable to the Latin American population. TOS is a tool to measure the tendency and interest in eating healthy food (HeOr) or pathological preoccupation with a rigid healthy diet (OrNe). Unlike all of these self-report questionnaires, the TON-17 is a more complex tool for assessing a broad spectrum of ON concept, based on current knowledge, criteria and state of art. The TON refers to the need to control food quality, worry about your own health, focus on a healthy lifestyle and high quality of life, obsessive behavior and intrusive thoughts about healthy eating, loss of previous interests and social relationships due to ON, loneliness, and deterioration of physical health. In this perspective, orthorexia appears to be the result of the tension between a healthy lifestyle attitude and a tendency towards compulsive behavior associated with a healthy diet.

The present study showed a strong construct validity using three structural analytic methods EFA, CFA and CCA. TON-17 may be considered to use in the future research, as a reasonably short, stable, reliable and valid instrument to ON measurement. Finally, this study makes a unique contribution to ON’s current understanding, showing that orthorexia can be a form of behavioral disorder, namely, healthy eating addiction. Addictive behaviors can span the entire spectrum of disorders, including more specific ones (e.g., EDs) and more general ones (e.g., OCD or anxiety). Addiction can also often lead to depression. More research is needed to address the classification problem of orthorexia.

### 4.3. Limitations of the Study

The present study has some limitations. Although a large sample size was involved in the present research, most of the participants were university students. Therefore, this convenience sample is not representative of the general population. The research sample is not homogeneous but consists of a number of different subgroups with different characteristics in terms of age, education, or gender distribution. Therefore, the results of the subgroups can only be compared to one another to a limited extent. Future research should be conducted in the general population to confirm the present psychometric properties and the structural and construct validity of the TON-17. Also, this study’s cross-sectional design does not allow to examine the dynamic aspect of ON and its development. In the future, a longitudinal study should be carried out. ORTO-15, used in this study for convergent validity, may indicate non-pathological orthorexic tendencies, which have been criticized [1,41]. Therefore, further studies using more appropriate ON measurement tools are needed to verify the convergent validation of the TON-17. BMI was calculated by the researchers in relation to the data reported by the participants in the questionnaire, which can be inaccurate measurement and could affect correlation. In the future, the analysis of association between ON and BMI should be derived from the objective and precise measurement of height and weight.

## 5. Conclusions

The TON-17 is a promising tool to assess the severity of orthorexia, comprising symptoms related to disordered eating, obsessive-compulsive disorder, and addiction to healthy food. Overall, this study confirmed the reliability and validity of the TON-17. The results of this study are consistent with previously established relationships between ON and demographic variables and various dimensions of physical and mental health. The TON-17 demonstrates good psychometric properties and stable bi-factor construction, with three primary and one secondary factors. More research is needed, especially with the use of various ON tools, to verify the findings.

The new measures may be useful in the treatment purposes. The multidimensional nature of ON requires the co-operation of psychotherapists, clinicians, and dieticians during treatment. Current best practices indicate that orthorexia could effectively be treated with a combination of cognitive-behavioral therapy, psychoeducation, and medication [11]. Strahler [43] suggested using cognitive-behavioral interventions focused on the food-related cognitions and beliefs regarding “healthy” eating.

## Figures and Tables

**Table 1:** Demographic characteristic of the study sample.

Demographic Variable	*n*	%	Range	*M*	*SD*
**Age**	767	100.00	18–78	26.49	9.66
**Height (cm)**	767	100.00	153–202	172.74	10.46
**Weight (kg)**	767	100.00	36.5–164	71.26	15.81
**Body Mass Index (BMI)**	767	100.00	15–67	23.71	4.32
BMI ≤ 18.5, underweight	40	5.22			
BMI 18.5–24.9, normal or healthy weight	492	64.15			
BMI 25.0–29.9, overweight	190	24.77			
BMI ≥ 30.0, obese	45	5.87			
**Gender**					
Women	437	56.98			
Men	327	42.63			
**Place of residence**					
Village	240	31.29			
City up to 20,000 residents	130	16.95			
City from 20,000 up to 100,000 residents	163	21.25			
City from 100,000 up to 500,000 residents	167	21.77			
City above 500,000 residents	63	8.21			
**Sample**					
Online	339	44.20			
Paper-and-pencil	428	55.80			
**Diet**					
I do not follow any special diet, I eat whatever I want	297	38.72			
I don’t follow any special diet, but I try to eat healthy	323	42.11			
Paleo diet (the basis is meat and animal fats)	6	0.78			
Meatless diet (vegetarian, vegetarian, vegan, fruitarian, etc.)	48	6.26			
A pure food diet (no artificial additives, preservatives, sugar, etc.)	17	2.22			
A color diet (the basis is fruits and vegetables of various colors)	4	0.52			
Box diet (ready-made meal sets developed by a dietitian)	10	1.30			
Slimming diet	24	3.13			
Other special diet	29	3.78			
**I use the current diet:**					
Because the doctor recommended me to	2	0.26			
To avoid unpleasant ailments (e.g., heartburn, stomach aches, headaches)	45	5.87			
For health	252	32.86			
I do not follow any special diet	446	58.15			
**Current food allergies or chronic diseases requiring a specific diet:**					
I do not have allergies or other chronic diseases that require a certain diet	601	78.36			
Celiac disease (gluten intolerance)	5	0.65			
Allergy to milk protein (casein, “protein blemish”) and/or lactose intolerance	51	6.65			
Other food allergy (e.g., to nuts, eggs, seafood)	35	4.56			
Non-food allergy (e.g., to nickel, pollen, dust)	38	4.95			
Type I diabetes	6	0.78			
Type II diabetes	7	0.91			
Hypertension	10	1.30			
Elevated cholesterol	13	1.69			
Reflux	22	2.87			
Stomach or duodenal ulcers	6	0.78			
Irritable bowel syndrome	24	3.13			
Gout	4	0.52			
Liver disease	2	0.26			
Other food-related disease	20	2.61			

**Table 2:** Fit indices for the TON-22, TON-23, and TON-17 factor models.

Sample											
EFA	CFA	Model	*n*	χ^2^/*df*	*p*	SRMR	RAMSEA (90% *CI*)	CFI	NFI	AIC	BIC
2	1	TON-22 One factor	256	5.01	<0.001	0.139	0.125 (0.118–0.131)	0.539	0.489	1138.951	1291.394
2	1	TON-22 Three-factor	256	2.83	<0.001	0.106	0.085 (0.077–0.093)	0.794	0.717	676.247	842.871
2	1	TON-22 Second-order	256	2.58	<0.001	0.12	0.079 (0.070–0.087)	0.834	0.759	18.114	834.370
2	1	TON-22 Bi-factor	256	1.91	<0.001	0.055	0.060 (0.050–0.069)	0.908	0.829	0.924	2.884
1	2	TON-23 One factor	256	6.07	<0.001	0.159	0.141 (0.134–0.148)	0.521	0.480	1490.949	1650.482
1	2	TON-23 Three-factor	256	2.44	<0.001	0.079	0.075 (0.064–0.086)	0.895	0.835	356.624	487.796
1	2	TON-23 Second-order	256	2.99	<0.001	0.139	0.076 (0.068–0.084)	0.875	0.809	649.986	891.058
1	2	TON-23 Bi-factor	256	2.37	<0.001	0.066	0.078 (0.070–0.086)	0.886	0.821	0.457	3.458
1/2	3	TON-17 One factor	255	7.62	<0.001	0.169	0.161 (0.152–0.171)	0.394	0.368	980.845	1097.707
1/2	3	TON-17 Three-factor	255	2.99	<0.001	0.097	0.088 (0.078–0.099)	0.824	0.76	420.736	551.763
1/2	3	TON-17 Second-order	255	3.21	<0.001	0.112	0.084 (0.078–0.190)	0.865	0.845	447.691	575.176
1/2	3	TON-17 Bi-factor	255	2.03	<0.001	0.050	0.053 (0.047–0.060)	0.949	0.927	0.549	0.871

Note. EFA = exploratory factor analysis, CFA = confirmatory factor analysis, SRMR = standardized root mean square residual, RAMSEA = root mean square error of approximation, CFI = comparative fit index, NFI = normed fit index, AIC = Akaike information criterion, BIC = Bayesian information criterion. *n* = 255 (Sample 3).

**Table 3:** The composite construct analysis results.

Scales and Items of the TON-17		Loading		Cronbach’s		
		F1, F2, F3	FG	α	AVE	CR
**General Factor (FG): Orthorexia**				0.79	0.24	0.84
**Factor 1 (F1): Control of food quality (*R*^2^ = 0.48)**				0.80	0.50	0.86
1	I am concerned about too much unhealthy food being available.	0.65	0.52			
2	I don’t trust food prepared by another person.	0.76	0.48			
3	Before I eat something, I make sure that the product has the appropriate health food quality certificates.	0.71	0.54			
4	I don’t eat GMO foods.	0.71	0.49			
5	I do not accept pesticide-produced foods in my diet.	0.71	0.35			
6	I often talk about healthy foods to convince others to change their diet.	0.72	0.51			
**Factor 2 (F2): Fixation on health and a healthy diet (*R*^2^ = 0.31)**				0.81	0.56	0.86
7	I pay a lot of attention to the ingredients of food I buy	0.80	0.52			
8	I plan each meal in detail.	0.80	0.58			
9	People who eat junk food are putting their lives at risk.	0.77	0.38			
10	Health is most important to me.	0.63	0.12			
11	Eating healthy food significantly affects my quality of life.	0.71	0.39			
**Factor 3 (F3): Disorder symptoms (*R*^2^ = 0.68)**				0.74	0.44	0.82
12	My diet makes me feel lonely.	0.70	0.62			
13	Due to the current diet, my health deteriorated.	0.56	0.41			
14	My relatives, doctors or other health care workers were concerned about my health condition and suggested that I change my diet.	0.56	0.37			
15	I pushed my hobbies and interests to the background by engaging in a healthy lifestyle.	0.75	0.60			
16	I prefer to eat a healthy meal alone than to go out with friends or family to eat something out.	0.66	0.59			
17	Food quality thoughts torment me most of the day.	0.69	0.61			

**Table 4:** Descriptive statistics for the TON-17 (Sample 3, *n* = 255).

			95% CI								Cronbach’sα	Correlations		
Scales	Range	*M*	*LL*	*UL*	*SD*	*SE*	*Mdn.*	Skew.	Kurt.	K-S *d*		TON-17	F1	F2
TON-17	17–79	42.17	41.44	42.90	10.34	0.37	42.00	0.55	0.62	0.06 **	0.76			
Factor 1	6–30	16.87	16.46	17.27	5.70	0.21	17.00	0.08	−0.79	0.06 **	0.80	0.71 ***		
Factor 2	5–25	14.83	14.49	15.17	4.83	0.17	15.00	−0.16	−0.80	0.09 **	0.81	0.62 ***	0.03	
Factor 3	6–29	10.47	10.17	10.78	4.31	0.16	9.00	1.48	2.58	0.15 **	0.73	0.76 ***	0.36 ***	0.32 ***

** *p* < 0.01, *** *p* < 0.001.

**Table 5:** Differences in ON between female and male participants, and people with a disease that require a restrictive diet and those without health problems.

	With Disease (*n* = 166)		Healthy (*n* = 601)				Women (*n* = 437)		Men (*n* = 327)			
TON-17	*M*	*SD*	*M*	*SD*	*t* (765)	*d*	*M*	*SD*	*M*	*SD*	*t* (762)	*d*
Total score (FG)	43.57	9.58	41.79	10.52	1.97	0.18	43.13	10.76	40.87	9.59	−3.00 **	0.22
Factor 1 (F1)	17.87	5.43	16.59	5.74	2.58 *	0.23	18.31	6.00	15.28	4.82	−6.76 ***	0.56
Factor 2 (F2)	14.81	5.10	14.84	4.76	−0.07	0.01	14.54	4.93	15.27	4.65	2.09 *	0.15
Factor 3 (F3)	10.89	4.40	10.36	4.29	1.40	0.12	10.58	4.56	10.32	3.97	−0.83	0.06

* *p* < 0.05, ** *p* < 0.01, *** *p* < 0.001.

**Table 6:** Correlations of the TON-17 scales (Total, F1, F2, and F3) with other variables.

Variable (Questionnaire)						TON-17			
	*N*	Range	*M*	*SD*	α	FG	F1	F2	F3
Orthorexia (ORTO-15)	182	15–51	35.58	5.97	0.65	0.34 ***	0.22 **	0.35 ***	0.19 *
Eating disorder (EAT-26)	339	0–65	9.96	9.18	0.86	0.36 ***	0.22 ***	0.32 ***	0.22 ***
Disordered eating attitude (DEAS)	481	41–135	78.35	15.60	0.62	0.31 ***	0.36 ***	0.02	0.26 ***
Relationship with food	481	12–58	22.74	6.80	0.50	0.19 ***	0.23 ***	−0.05	0.20 ***
Concerns about food and weight	481	3–20	7.33	3.07	0.14	0.26 ***	0.33 ***	0.02	0.19 ***
Restrictive practices	481	3–20	9.46	5.03	0.30	0.11 *	0.25 ***	−0.15 **	0.11 *
Feeling toward eating	481	2–15	5.34	2.78	0.02	0.13 **	0.17 ***	−0.12 **	0.23 ***
Idea of normal eating	481	4–20	11.85	4.53	0.44	0.30 ***	0.21 ***	0.29 ***	0.15 **
Health behavior (HBI)	155	40–113	78.35	14.33	0.84	0.47 ***	0.48 ***	0.49 ***	0.21 **
Healthy Eating Habits (HEH)	155	7–30	19.37	5.40	0.85	0.59 ***	0.63 ***	0.60 ***	0.25 **
Preventive Behavior (PB)	155	7–30	18.81	4.85	0.69	0.33 ***	0.39 ***	0.32 ***	0.12
Positive Mental Attitude (PMA)	155	9–52	20.86	4.69	0.52	0.18 *	0.18 *	0.21 **	0.07
Healthy Practices (HP)	155	10–29	19.30	4.05	0.58	0.27 **	0.18 *	0.30 ***	0.20 *
Body mass index (BMI)	767	15–67	23.71	4.32	0.63	0.07 *	<0.01	0.06	0.10 **
Quality of life (WHOQOL)	329	55–130	98.16	14.57	0.92	0.04	−0.02	0.20 ***	−0.11
Physical	329	12–35	26.46	4.40	0.81	0.03	−0.03	0.17 **	−0.08
Psychological	329	6–30	22.95	4.06	0.85	0.02	−0.03	0.21 ***	−0.15 **
Social Relationship	329	4–15	12.09	2.20	0.76	0.04	0.02	0.12 *	−0.06
Environment	329	16–40	29.28	4.22	0.73	0.10	0.04	0.17 *	<0.01
Physical health (GSRH)	674	2–10	5.13	1.60	0.80	−0.05	0.02	−0.18 ***	0.07
Obsessive-compulsive disorder (OCIR)	339	0–64	20.26	14.02	0.91	0.44 ***	0.26 ***	0.25 ***	0.46 ***
Washing	339	0–12	2.80	2.92	0.76	0.40 ***	0.26 ***	0.21 ***	0.41 ***
Checking	339	0–12	2.62	3.05	0.85	0.34 ***	0.28 ***	0.17 **	0.26 ***
Ordering	339	0–12	4.23	2.87	0.59	0.38 ***	0.25 ***	0.22 ***	0.34 ***
Obsessing	339	0–12	4.21	3.23	0.74	0.32 ***	0.09	0.21 ***	0.44 ***
Hoarding	339	0–12	4.39	3.28	0.77	0.27 ***	0.10	0.20 ***	0.31 ***
Neutralizing	339	0–12	2.01	2.69	0.75	0.34 ***	0.20 ***	0.14 **	0.41 ***
Generalized anxiety disorder (GAD-7)	642	0–21	5.90	5.25	0.92	0.12 **	0.09 *	−0.02	0.19 ***
Depression (PHQ-9)	219	0–23	6.74	4.99	0.84	0.19 **	0.19 **	−0.12	0.33 ***

* *p* < 0.05, ** *p* < 0.01, *** *p* < 0.001.

## Data Availability

The data presented in this study are available on request from the corresponding author.

## Supplementary Materials

The following are available online at https://www.mdpi.com/article/10.3390/jcm10081637/s1, TON-17_EN: Test of Orthorexia Nervosa in English, TON-17_PL: Test of Orthorexia Nervosa in Polish, Tables S1–S8: Table S1. Structured interview for orthorexia research, Table S2. Samples in the study, Table S3. Reliability and item analysis of the TON-40, Table S4. Test–re-test reliability (*n* = 126), Table S5: Table S5a. Inter-item correlations for TON-40 (Sample 1, *n* = 256), Table S5b. Inter-item correlations for TON-40 (Sample 2, *n* = 256). Table S6. Comparison of EFA 1, EFA2, and EFA 3 results., Table S7. Inter-item correlations for TON-17 (Sample 3, *n* = 255), Table S8. Reliability and item analysis of the TON-17 (Sample 3, *n* = 255).
