# Supplementary material for: Development and Validation of the Test of Orthorexia Nervosa (TON-17)
Source: J Clin Med. 2021 Apr 12;10(8):1637. doi: 10.3390/jcm10081637 (PMC8069172; doi:10.3390/jcm10081637)
Supplement: Supplementary file 1 [file jcm-10-01637-s001.zip › Supplementary Materials/Supplementary Materials_Tables_S1-S8.pdf]

Supplementary Materials: Tables S1 – S8

Table S1. Structured interview for orthorexia research

**Healthy lifestyle**
1. What does healthy lifestyle mean for you?
2. When did you first start to be interested in healthy food and lifestyle?
3. What made you pay more attention to what you eat (friends, authorities, family, sickness, media)?
4. Did you seek information from medical professionals, dieticians, or did you search for information on healthy eating on the Internet?
5. What diets have you tried so far? Why?
6. Do you exercise? How often? What kind of exercises do you do? For how long? Does your mood depend on that? What impact does exercise have on your diet?
7. Do you control your weight? To what extent does your weight determine your well-being/self-esteem?
8. Do you count calories before deciding to eat? How often does this happen?
9. Have you ever restricted the amount of food you eat to a minimum to achieve a perfect figure? How long did it last?
10. Have you ever eaten a lot of food at once and then forced vomiting? How often? For how long?
**Family and relationships**
1. What was the diet like in your family home?
2. Has anyone in your family had symptoms of diseases such as: diabetes, reflux, stomach ulcers, lactose intolerance, celiac disease, allergy?
3. Has anyone in your family ever restricted the amount of food they eat to a minimum to achieve a perfect figure? How long did it last?
4. Has anyone from your family ever eaten a lot of food at once and then forced vomiting? How often? For how long?
5. Can you depend on your family in case of trouble? Describe such a situation.
6. Do your friends and family support you in maintaining your diet?
7. Does your way of eating affect your life (selection of the job, place of work, friends, contacts with family)?
8. Do you feel lonely because people don't understand your lifestyle?
9. Do you talk with other people about healthy diet? How often?
10. What is your opinion about the people who do not pay attention to the quality of the food they consume?
**Current diet**
1. Describe your current diet. What do you eat? How often? What products do you avoid? For how long have you been following your current diet?
2. Describe your process of planning and preparing your food. What do you think of while doing this? How does it make you feel?

3. Where do you buy your food?
4. Before buying a product, do you check
1. for preservatives?
2. for artificial food coloring?
3. country of origin?
4. bio/organic/eco certificates?
5. amounts of sugars, sodium, fats, vitamins, and proteins?
5. What is important for you when choosing food (taste, price, ingredients, appearance)?
6. Do you often find yourself not being able to stop thinking about the quality of the food you eat? How often? Does it bother you? How much?
7. Does maintaining your current diet make you feel more in control of yourself, your body, and your life?
**Current health**
1. Has your condition changed after starting the diet (for better or worse)?
2. How would you rate your health right now?
3. Do you have symptoms of such diseases as: diabetes, reflux, stomach ulcers, lactose intolerance, celiac disease, allergy?
4. Have you recently blacked out, fainted, lost consciousness? When? How often does it happen? How many times did it happen in the last year?
5. What is health for you? Where would you put your health on your priority list?
6. What makes you happy? What determines your wellbeing?
7. What is healthy food for you?

Table S2. Samples in the study

Sample	Form of survey	Recruiting method	*n*	*n*(%) of Women	M_{Age}	SD_{Age}	TON-40	ORTO-15	EAT-26	DEAS	HBI	WHOQO-	GSRH	OCIR	GAD-7	PHQ-9
1	On-line	Snowball: mailing list, Facebook	182	77(42%)	27.04	8.24	*	*	*				*	*	*	
2	Paper-pencil	Snowball: occasional meetings	157	95(61%)	31.99	13.79	*		*	*	*		*	*	*	
3	Paper-pencil	Psychology students at University	133	111(83%)	20.75	1.27	*			*		*	*		*	*
4	Paper-pencil	Health care staff from hospital and soldiers from one military unit	82	33(40%)	33.39	10.71	*			*		*			*	
5	Paper-pencil	Students at university	121	121(100%)	24.06	4.97	*			*		*	*			
6	Paper-pencil	Physical activity students	92	0(0%)	21.10	2.05	*						*		*	*

Table S3. Reliability and item analysis of the TON-40

Item No.	Statistics if the item deleted				Item-Total
	M	S^2	*SD*	α	*r*
1.	87.92	628.66	25.07	0.932	0.41
2.	88.97	632.82	25.16	0.931	0.45
3.	87.48	632.80	25.16	0.931	0.43
4.	88.81	629.19	25.08	0.931	0.45
5.	87.96	630.30	25.11	0.931	0.47
6.	89.08	638.46	25.27	0.932	0.40
7.	88.59	629.34	25.09	0.931	0.49
8.	87.72	642.39	25.35	0.933	0.24
9.	88.76	633.47	25.17	0.931	0.45
10.	88.44	623.25	24.96	0.930	0.59
11.	89.07	627.13	25.04	0.930	0.58
12.	88.68	623.39	24.97	0.930	0.63
13.	88.18	631.41	25.13	0.931	0.47
14.	89.14	629.49	25.09	0.930	0.56
15.	88.82	630.19	25.10	0.931	0.52
16.	89.03	631.27	25.13	0.931	0.45
17.	88.20	633.16	25.16	0.932	0.39
18.	88.86	629.35	25.09	0.930	0.53
19.	89.01	629.31	25.09	0.930	0.55
20.	88.41	626.05	25.02	0.931	0.49
21.	89.05	622.30	24.95	0.929	0.68
22.	89.54	634.52	25.19	0.930	0.58
23.	89.05	626.73	25.03	0.930	0.55
24.	89.20	612.68	24.75	0.934	0.39
25.	89.07	631.52	25.13	0.931	0.47
26.	89.41	633.31	25.17	0.930	0.54
27.	88.45	627.31	25.05	0.931	0.49
28.	88.46	629.98	25.10	0.931	0.43
29.	89.23	637.30	25.24	0.932	0.39
30.	89.01	618.04	24.86	0.929	0.73
31.	88.97	622.29	24.95	0.929	0.66
32.	89.54	633.01	25.16	0.930	0.60
33.	88.04	634.10	25.18	0.932	0.36
34.	88.68	624.92	25.00	0.930	0.60
35.	88.82	623.71	24.97	0.930	0.61
36.	89.37	630.99	25.12	0.930	0.59
37.	89.27	630.04	25.10	0.930	0.57
38.	88.96	633.25	25.16	0.932	0.40
39.	89.16	637.17	25.24	0.932	0.38
40.	89.06	625.61	25.01	0.930	0.61

Table S4. Test–re-test reliability (n = 126).

Item of TON-40	*M*	*SD*	*M*	*SD*	Cohen's Kappa	Pearson's *r*	Spearman's *r*
1.	4.16	0.89	3.83	0.98	0.40	0.60	0.63
2.	2.48	1.06	2.48	1.03	0.48	0.71	0.69
3.	3.56	1.13	3.13	1.16	0.21	0.59	0.58
4.	3.02	1.29	2.83	1.29	0.39	0.72	0.72
5.	3.25	1.25	2.94	1.25	0.41	0.74	0.73
6.	2.20	1.14	2.21	1.13	0.23	0.43	0.45
7.	2.05	0.95	1.94	0.91	0.30	0.53	0.54
8.	3.67	0.99	3.76	1.08	0.36	0.60	0.63
9.	2.02	0.98	1.81	0.97	0.32	0.57	0.55
10.	2.79	1.17	2.58	1.20	0.35	0.60	0.60
11.	1.52	0.78	1.43	0.65	0.32	0.38	0.40
12.	2.17	0.92	2.00	0.95	0.36	0.51	0.53
13.	2.94	1.12	2.79	1.11	0.28	0.60	0.59
14.	1.94	1.02	1.83	0.86	0.32	0.41	0.43
15.	1.80	0.95	1.73	0.97	0.34	0.45	0.48
16.	2.52	1.13	2.36	1.26	0.37	0.55	0.51
17.	3.73	1.05	3.20	1.18	0.21	0.55	0.58
18.	2.33	1.11	2.38	1.06	0.41	0.62	0.60
19.	1.84	0.93	1.70	0.88	0.40	0.63	0.52
20.	1.94	1.03	1.89	1.02	0.29	0.64	0.58
21.	2.04	1.01	2.03	1.03	0.33	0.48	0.47
22.	1.43	0.79	1.48	0.92	0.21	0.30	0.25
23.	2.28	1.40	2.33	1.32	0.39	0.61	0.59
24.	1.47	0.77	1.46	0.73	0.40	0.48	0.47
25.	1.21	0.59	1.33	0.74	0.26	0.37	0.30
26.	1.21	0.64	1.29	0.67	0.23	0.21	0.30
27.	2.14	1.15	2.30	1.19	0.33	0.57	0.61
28.	1.85	1.00	1.79	0.95	0.29	0.60	0.53
29.	1.90	1.26	1.85	1.21	0.26	0.63	0.53
30.	2.06	1.09	2.10	1.14	0.39	0.64	0.67
31.	1.97	1.02	1.91	0.92	0.33	0.55	0.51
32.	1.34	0.62	1.40	0.71	0.28	0.45	0.33
33.	2.39	1.11	2.22	1.09	0.32	0.58	0.58
34.	2.36	1.11	2.26	1.12	0.36	0.65	0.65
35.	2.29	1.08	2.19	1.01	0.36	0.61	0.61
36.	1.65	0.96	1.70	0.90	0.43	0.63	0.63
37.	1.85	1.10	1.83	0.96	0.39	0.62	0.59
38.	2.42	1.55	2.44	1.46	0.35	0.67	0.63
39.	1.57	0.92	1.74	0.96	0.45	0.56	0.54
40.	1.69	0.93	1.81	1.00	0.28	0.63	0.51
M	2.23	1.02	2.16	1.02	0.34	0.56	0.54

Table S6. Comparison of EFA 1, EFA2, and EFA 3 results.

Item	Content	EFA 1 Sample 1 (n = 256)			EFA 2 Sample 2 (n = 256)			EFA 3 Sample 3		
		**F1**	**F2**	**F3**	**F1**	**F2**	**F3**	**F1**	**F2**	**F3**
1	I am concerned about too much unhealthy food being available.	**0.44**			**0.57**			0.51		
2	I pay a lot of attention to the composition of the food I buy.		**0.57**			**0.64**			0.73	
3	I don't trust food prepared by another person.	**0.66**			**0.69**			0.67		
4	Before I eat something, I make sure that the product has the appropriate health food quality certificates.	**0.60**			**0.61**			0.55		
5	I don't eat GMO foods.	**0.70**			**0.71**			0.63		
6	I always check the country of origin of a food product.					0.37			0.39	
7	I exclude artificially enhanced products (containing synthetic pigments, flavor and aroma enhancers) from my diet.								0.66	
8	I do not accept pesticide-produced foods in my diet.	**0.73**			**0.71**			0.71		
9	Foods containing artificial preservatives are inedible.					0.52				
10	I spend a lot of time preparing food.				0.46				0.29	
11	I plan each meal in detail.		**0.55**			**0.64**			0.65	
12	People are usually unable to accept my diet.	0.48								
13	I avoid social gatherings because I am afraid there will be no healthy food there.	0.71								
14	I am forced to take my food to meetings with friends.									
15	I constantly think about healthy food.			0.46						
16	I give up eating when I don't have healthy food within reach.				0.51					
17	I often talk about healthy foods to convince others to change their diet.	**0.68**			**0.66**					
18	Junk food disgusts me.									
19	People who eat junk food are putting their lives at risk.		**0.69**			**0.76**			0.76	
20	At every opportunity, I encourage others to change their diet.									
21	I don't meet people who eat unhealthy food.									
22	My diet makes me feel lonely.			0.67			0.71			0.57
23	People tell me that my health has deteriorated.									0.43

24	I feel guilty when I eat something unhealthy.								0.59	
25	The health value of a product is more important to me than its taste.		0.70						0.72	
26	I keep my diet up to date for the sake of my health.		0.62						0.72	
27	Health is most important to me.		**0.69**			**0.62**			0.67	
28	Due to the current diet, my health deteriorated.			**0.57**			**0.58**			0.46
29	My relatives, doctors or other health care workers were concerned about my health condition and suggested that I change my diet.			**0.55**			**0.66**			0.54
30	I feel a constant need to look for healthy food products.									
31	Trying to restrict healthy foods in my diet causes me irritation, depression, or annoyance.									
32	I pushed my hobbies and interests to the background by engaging in a healthy lifestyle.			0.61			0.66			0.64
33	My friends complain that I care too much about a healthy lifestyle.									
34	Life without healthy food is empty and meaningless.	0.50								
35	It bothers me when someone gives me unhealthy food to eat.									
36	I prefer to eat a healthy meal alone than to go out with friends or family to eat something out.			**0.58**			**0.58**			0.48
37	Food quality thoughts torment me most of the day.			**0.60**			**0.57**			0.51
38	Eating healthy food significantly affects my quality of life.		**0.61**			**0.65**			0.61	
39	I exclude certain products from my diet (e.g. sugar, gluten, lactose), even though I am not allergic to them.					0.57			0.41	
40	I don't eat meat, eggs or dairy products if they contain antibiotics or hormones.		0.47							

Table S7. Inter-item correlations for TON-17 (Sample 3, n = 255).

Item	1	2	3	4	5	6	7	8	9	10	11	12	13	14	15	16	17
1	1.00																
2	0.32***	1.00															
3	0.39***	0.05	1.00														
4	0.28***	0.22***	0.46***	1.00													
5	0.33***	0.06	0.41***	0.44***	1.00												
6	0.33***	-0.05	0.51***	0.35***	0.55***	1.00											
7	0.22***	0.52***	0.04	0.14*	0.13*	-0.04	1.00										
8	0.39***	0.09	0.48***	0.41***	0.31***	0.44***	0.11	1.00									
9	0.11	0.52***	-0.19**	0.03	-0.01	-0.24***	0.45***	-0.12	1.00								
10	0.14*	0.15*	0.19**	0.21**	0.23***	0.17**	0.38***	0.22***	0.16*	1.00							
11	-0.10	0.39***	-0.36***	-0.15*	-0.12	-0.34***	0.40***	-0.27***	0.60***	0.03	1.00						
12	0.08	0.14*	0.06	0.10	0.08	-0.04	0.11	0.06***	0.18**	0.29***	0.13*	1.00					
13	0.04	0.02	0.17**	0.13*	0.03	0.06	0.03	0.14*	0.07	0.32***	-0.11	0.40***	1.00				
14	0.09	0.19**	0.19**	0.10	0.09	0.10	0.40***	0.21**	0.16*	0.49***	0.06	0.30***	0.27***	1.00			
15	0.16*	0.18	0.21**	0.22***	0.23***	0.12	0.24***	0.27***	0.17**	0.34***	0.05	0.15*	0.25***	0.42***	1.00		
16	0.26***	0.20**	0.21**	0.25***	0.17**	0.10	0.25***	0.28***	0.18**	0.33***	0.00	0.30***	0.26***	0.40***	0.36***	1.00	
17	0.14*	0.40***	-0.10	0.11	0.09	-0.19**	0.39***	-0.06	0.52***	0.11	0.46***	0.20**	-0.02	0.16*	0.20**	0.15*	1.00

*$p < 0.05$, **$p < 0.01$, ***$p < 0.001$

Table S8. Reliability and item analysis of the TON-17 (Sample 3, *n* = 255)

Item No.	Statistics if the item deleted				Item-Total
	M	S^2	*SD*	α	*r*
1.	38.01	80.14	8.95	0.76	0.43
2.	38.49	79.43	8.91	0.76	0.47
3.	39.08	81.23	9.01	0.77	0.34
4.	39.14	80.17	8.95	0.76	0.44
5.	39.06	80.71	8.98	0.76	0.40
6.	38.57	83.83	9.16	0.78	0.22
7.	39.25	79.10	8.89	0.75	0.50
8.	38.89	80.42	8.97	0.76	0.37
9.	38.68	80.94	9.00	0.77	0.34
10.	40.04	83.19	9.12	0.76	0.48
11.	38.20	87.05	9.33	0.79	0.09
12.	39.52	83.93	9.16	0.77	0.32
13.	39.66	84.65	9.20	0.77	0.23
14.	40.00	82.91	9.11	0.76	0.45
15.	39.89	82.58	9.09	0.76	0.45
16.	39.66	81.04	9.00	0.76	0.47
17.	38.31	80.07	8.95	0.77	0.34

.
