# Supplementary material for: Development and Validation of the Test of Orthorexia Nervosa (TON-17)
Source: J Clin Med. 2021 Apr 12;10(8):1637. doi: 10.3390/jcm10081637 (PMC8069172; doi:10.3390/jcm10081637)
Supplement: Supplementary file 1 [file jcm-10-01637-s001.zip › Supplementary Materials/Supplementary Materials_TON-17_EN.pdf]

TON-17

Rogowska, Kwaśnicka, & Ochnik, 2021

Below are some statements that may describe your condition, feelings and behavior. Please read each statement carefully and then select the answer that best reflects your feelings and behavior. Please circle your answer on the scale that shows the degree of compliance with the statement:

**1 = Strongy disagree**<br>**2 = Rather disagree**<br>**3 = Undecided**<br>**4 = Rather agree**<br>**5 = Strongly agree**		**Strongy disagree**	**Rather disagree**	**Undecided**	**Rather agree**	**Strongly agree**
1.	I am concerned about too much unhealthy food being available.	1	2	3	4	5
2.	I don't trust food prepared by another person.	1	2	3	4	5
3.	Before I eat something, I make sure that the product has the appropriate health food quality certificates.	1	2	3	4	5
4.	I don't eat GMO foods.	1	2	3	4	5
5.	I do not accept pesticide-produced foods in my diet.	1	2	3	4	5
6.	I often talk about healthy foods to convince others to change their diet.	1	2	3	4	5
7.	I pay a lot of attention to the ingredients of food I buy	1	2	3	4	5
8.	I plan each meal in detail.	1	2	3	4	5
9.	People who eat junk food are putting their lives at risk.	1	2	3	4	5
10.	Health is most important to me.	1	2	3	4	5
11.	Eating healthy food significantly affects my quality of life.	1	2	3	4	5
12.	My diet makes me feel lonely.	1	2	3	4	5
13.	Due to the current diet, my health deteriorated.	1	2	3	4	5
14.	My relatives, doctors or other health care workers were concerned about my health condition and suggested that I change my diet.	1	2	3	4	5
15.	I pushed my hobbies and interests to the background by engaging in a healthy lifestyle.	1	2	3	4	5
16.	I prefer to eat a healthy meal alone than to go out with friends or family to eat something out.	1	2	3	4	5
17.	Food quality thoughts torment me most of the day.	1	2	3	4	5
	Summarize all selected scores in the columns =					
