# Supplementary material for: Development and Validation of the Test of Orthorexia Nervosa (TON-17)
Source: J Clin Med. 2021 Apr 12;10(8):1637. doi: 10.3390/jcm10081637 (PMC8069172; doi:10.3390/jcm10081637)
Supplement: Supplementary file 1 [file jcm-10-01637-s001.zip › Supplementary Materials/Supplementary Materials_TON-17_PL.pdf]

TON-17

Rogowska, Kwaśnicka, & Ochnik, 2021

Poniżej znajdują się twierdzenia, które służą do samodzielnego opisania swojego stanu, odczuć i zachowań. Proszę dokładnie przeczytać każde stwierdzenie, a następnie zaznaczyć kółkiem odpowiedź, która najlepiej odwzorowuje Pana/Pani odczucia i zachowania, zgodnie ze skalą 1-5, gdzie:

1 = Zdecydowanie nie zgadzam się<br>2 = Raczej nie zgadzam się<br>3 = Nie mam zdania<br>4 = Raczej zgadzam się<br>5 = Zdecydowanie zgadzam się		Zdecydowanie nie zgadzam się	Nie zgadzam się	Nie mam zdania	Zgadzam się	Zdecydowanie zgadzam się
1.	Niepokoi mnie zbyt duża dostępność niezdrowego jedzenia.	1	2	3	4	5
2.	Nie ufam żywności przygotowanej przez kogoś innego.	1	2	3	4	5
3.	Zanim coś zjem, upewniam się, czy dany produkt posiada odpowiednie certyfikaty jakości zdrowej żywności.	1	2	3	4	5
4.	Nie jadam żywności GMO.	1	2	3	4	5
5.	Nie akceptuję w swojej diecie żywności wyprodukowanej przy pomocy pestycydów.	1	2	3	4	5
6.	Często podejmuję rozmowy na temat zdrowej żywności, żeby przekonać innych do zmiany sposobu odżywiania.	1	2	3	4	5
7.	Zwracam dużą uwagę na skład kupowanej żywności.	1	2	3	4	5
8.	Szczegółowo planuję każdy posiłek.	1	2	3	4	5
9.	Osoby spożywające śmieciową żywność narażają swoje życie.	1	2	3	4	5
10.	Zdrowie jest dla mnie najważniejsze.	1	2	3	4	5
11.	Spożywanie zdrowego jedzenia znacząco wpływa na jakość mojego życia.	1	2	3	4	5
12.	Mój sposób żywienia powoduje, że czuję się samotny/samotna.	1	2	3	4	5
13.	W związku z aktualnym sposobem odżywiania, mój stan zdrowia pogorszył się.	1	2	3	4	5
14.	Moi bliscy, lekarze lub inni pracownicy opieki zdrowotnej byli zaniepokojeni moim stanem zdrowia i sugerowali mi zmianę sposobu odżywiania.	1	2	3	4	5
15.	Zepchnąłem/zepchnęłam swoje hobby i zainteresowania na drugi plan przez moje zaangażowanie w zdrowy styl życia.	1	2	3	4	5
16.	Wolę spożyć w samotności zdrowy posiłek niż wyjść ze znajomymi lub rodziną żeby zjeść coś na mieście.	1	2	3	4	5
17.	Przez większą część dnia dręczą mnie myśli o jakości spożywanego jedzenia.	1	2	3	4	5
	Proszę dodać do siebie zaznaczone wyniki w kolumnach =					
